# Plasma pharmacokinetics of tigolaner, emodepside, and praziquantel following topical administration of a combination product (Felpreva®) and of intravenous administration of the individual active ingredients in cats

**DOI:** 10.1016/j.crpvbd.2023.100126

**Published:** 2023-06-22

**Authors:** Norbert Mencke, Wolfgang Bäumer, Kristine Fraatz, Ralph Krebber, Marc Schneider, Katrin Blazejak

**Affiliations:** a Vetoquinol, Department of Medical Marketing Parasitology, 37 rue de la Victoire, 75009 Paris, France; b Institute of Pharmacology and Toxicology, Department of Veterinary Medicine, Freie Universität Berlin, Berlin, Germany; c Elanco Animal Health Company, Alfred Nobel Str. 50, 40789 Monheim, Germany; d Bayer AG, Crop Science Division, 40789 Monheim am Rhein, Germany; e Vetoquinol Bioanalysis and Pharmacokinetic, Development, Lure, France

**Keywords:** Tigolaner, Emodepside, Praziquantel, Cat, Pharmacokinetics, Felpreva®

## Abstract

Felpreva® for cats contains the new acaricidal/insecticidal active ingredient tigolaner in a fixed combination with the nematocidal and cestocidal compounds emodepside and praziquantel, respectively. The plasma pharmacokinetics of tigolaner, emodepside, and praziquantel were evaluated in clinically healthy cats following topical (spot-on) treatment as fixed combination Felpreva®. For the determination of bioavailability intravenous administration of single active ingredients was also performed. After a single topical administration of Felpreva® using the target dose volume of 0.148 ​ml/kg to cats, tigolaner reached mean peak concentrations of 1352 ​μg/l with a T_max_ of 12 days and a mean half-life of 24 days. Simulation of repetitive topical administration every 91 days indicates only a low risk of accumulation after reaching steady state within two to three administrations. The volume of distribution calculated after intravenous dosing was 4 ​l/kg and plasma clearance was low with 0.005 ​l/h/kg. Overall plasma exposure was 1566 ​mg∗h/l after topical administration, providing an absolute bioavailability of 57%. Tigolaner was mainly cleared *via* the faeces (54% within 28 days), renal clearance was neglectable (< 0.5% within 28 days). Emodepside and praziquantel showed mean peak concentrations of 44 ​μg/l and 48 ​μg/l (reached after 1.5 days and 5 ​h, respectively). Overall plasma exposures were 20.6 and 3.69 ​mg∗h/l, respectively. The elimination half-life was 14.5 days for emodepside and 10 days for praziquantel after topical administration. After topical administration of Felpreva® using 2.5× and 5× dose multiples an almost proportional increase of plasma exposure was observed for all three active ingredients. With the addition of tigolaner, Felpreva® combines the established pharmacokinetic (PK) characteristics of emodepside and praziquantel contained in Profender® spot-on for cats with the favourable PK of tigolaner suitable for a 3-months protection against fleas and ticks.

## Highlights

• First pharmacokinetic data about the three active ingredients of Felpreva® (tigolaner, praziquantel and emodepside) in cats.
• Tigolaner displays long half-life and high volume of distribution after topical administration.
• Up to 5 times the recommended dose of Felpreva® is well tolerated in cats.
• Emodepside and praziquantel pharmacokinetic profiles ensure efficacy comparable to Profender®.

## Graphical abstract

**Figure:**
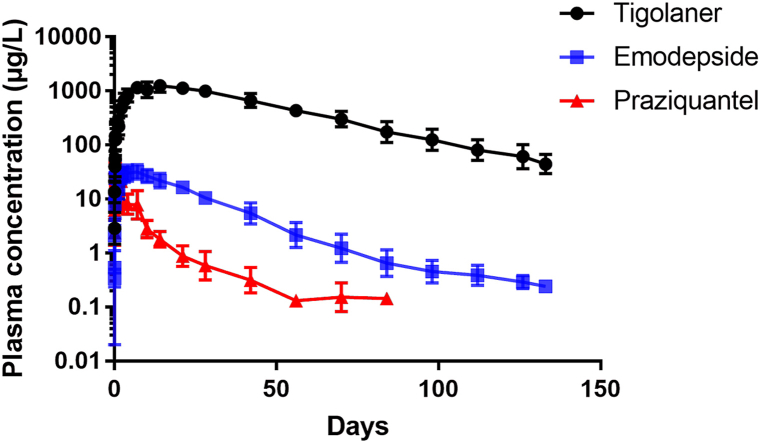

## 1 Introduction

Felpreva® containing tigolaner as a new active ingredient (AI), in combination with the well-established nematocide emodepside and the cestocide praziquantel is a new commercially available treatment and protection against infestations with fleas (*Ctenocephalides felis*), ticks (*Ixodes ricinus*, *Ixodes holocyclus*) and mites (*Notoedres cati*, *Otodectes cynotis*), as well as infections with lungworms (*Aelurostrongylus abstrusus*, *Troglostrongylus brevior*), gastrointestinal nematodes (*Toxocara cati*, *Toxascaris leonina*, *Ancylostoma tubaeforme*) and cestodes (*Dipylidium caninum*, *Taenia taeniaeformis*), providing safe, rapid and long-acting efficacy in cats following a single spot-on administration. Felpreva is indicated when ectoparasites, cestodes and nematodes are to be treated at the same time. The volume to apply dermally is 0.37 ​ml for a small cat (1.0–2.5 ​kg), 0.74 ​ml for medium-sized cats (2.6–5.0 ​kg) and 1.18 ​ml for large cats (5.1–8.0 ​kg). Felpreva is licensed in Europe since November 2021.

Tigolaner, from the bis-pyrazole class of compounds has potent antiparasitic properties acting against γ-aminobutyric acid- (GABA-) and glutamate-gated chloride channels with significant selectivity for insect neurons over mammalian neurons (EMA, 2022). Tigolaner has a high potency against insects and acarids by exposure *via* their feeding, i.e. fleas and ticks that initiate feeding will be exposed to the AI (Cvejić et al., 2022a, b; Mencke et al., 2023). A single tigolaner dose administered topically at the minimum recommended dose of 14.5 ​mg/kg body weight (BW) on cats provides 13 weeks of flea and tick control (Cvejić et al., 2022a, b; Mencke et al., 2023). A fast onset of flea efficacy, the so called speed of flea kill is of clinical relevance to reduce exposure to flea saliva (flea allergic dermatitis) and transmission of pathogens (vector-borne diseases). Studies clearly demonstrated the fast onset of efficacy within 12 ​h with respect to fleas that are already on the cat prior to treatment. For new flea infestation the onset was within 8 ​h for two months and within 24 ​h afterwards (EMA, 2022; Mencke et al., 2023). The fast onset together with the long duration of activity after a single topical administration offers a convenient alternative to monthly flea and tick control treatments and is expected to increase pet owner compliance (Lavan et al., 2020, 2021). Increased compliance assists in limiting protection gaps that can occur with missed re-administration of monthly treatments. Felpreva® is effective in treatment of an existing infestation with ear (*Otodectes cynotis*) and head mange (*Notoedres cati*) mites in a single spot-on application (EMA, 2022) and prevent tick paralysis caused by *Ixodes holocyclus* (Roeber et al., 2023).

Emodepside and praziquantel are already combined in Profender® spot on for cats, which is a well-established helminth protection and treatment with a broad spectrum of activity against both nematodes and cestodes (Altreuther et al., 2005; Taweethavonsawat et al., 2013). With the addition of tigolaner in Felpreva® the therapeutic range is extended to a reliable flea and tick control (Cvejić et al., 2022a).

The present study focusses on the pharmacokinetic (PK) profile of tigolaner, emodepside and praziquantel in cats following a single topical administration of a fixed combination (Felpreva®) with regard to the absorption, distribution, metabolism and elimination. Additionally, it provides insight into possible dose proportionality of the AIs. In addition, a simulation of repeated tigolaner administration (every 91 days) was performed to reveal information about possible cumulation potential. This is of particular interest, as 8-week intervals reveal a possible cumulative behaviour of tigolaner, leading to the Summary of Product Characteristics (SPC) advice that the product should not be administered at intervals shorter than 8 weeks.

## 2 Materials and methods

### 2.1 Overview of studies

Altogether three studies were conducted. Cats were treated with Felpreva® (containing emodepside praziquantel and tigolaner) at the recommended dose (14.5 ​mg/kg tigolaner, 3 ​mg/kg emodepside and 12 ​mg/kg praziquantel) or at 2.5× and 5× the recommended dose. For intravenous (i.v.) injection (Study 1) tigolaner, emodepside and praziquantel were formulated in tetraglycol as 8.9%, 1.85% and 7.4% solutions, respectively.

In each study all animal husbandry and study conduct were compliant with local regulations including the Directive 2010/63/EU of the European Parliament and of the Council of 22nd September 2010 on the protection of animals used for scientific purposes. Studies 1 and 2 were performed in Germany and the study design and the experimental procedures had been approved by the responsible authorities (LANUV - Regional authority for nature, environment and consumer protection in North Rhine Westphalia). Study 3 was performed in the Netherlands and approved by the Central Authority for Scientific Procedures on Animals (CCD) as required by the Dutch Act on Animal Experimentation.

### 2.2 *In vivo* phase

Table 1 provides animal details for each of the 3 studies and Table 2 presents the study designs. All cats were healthy and acclimatized for a minimum of 7 days. In studies 1 and 2, cats were individually housed for 8 ​h or 10–12 days following i.v. or topical administration, respectively, to avoid potential cross-contamination between animals. After this period, cats were group-housed by treatment group and sex. In Study 3, cats were socially housed in groups of 3 (same sex/same dose group) in one or more connected similar cages. The exception was when cats were separated for designated study procedures/activities associated with dosing or urine and faeces collection.

**Table 1:** Description of cats in each study.

Study	No. of cats	Cat breed	Cat age (months)	Sex	Body weight (kg)
1	28	DSH	20–23	16M; 12F desexed	3.2–6.0
2	16	ESH	16–30	8M; 8F desexed	3.7–6.6
3	6	DSH	12–36	3M; 3F	2.7–5.0

*Abbreviations*: DSH, Domestic Shorthair; ESH, European Shorthair; M, male; F, female.

**Table 2:** Study designs.

Group	No. of treated cats	Dosage	Blood sampling times (hours)^a^
Study 1			
Tigolaner	6	1.5 ​mg/kg i.v.	(day −5) (3 and 6 ​min) 0.25, 0.5, 1, 2, 4, 8, 24, 48, 72, 96, 168, 240, 336, 504, 672, 1008, 1344, 1680, 2016, 2352, 2688, 3024, 3360
Emodepside	6	0.3 ​mg/kg i.v.	(day −5) (3 and 6 ​min) 0.25, 0.5, 1, 2, 4, 8, 24, 48, 72, 96, 168, 240, 336, 504, 672, 1008, 1344, 1680, 2016, 2352
Praziquantel	6	0.2 ​mg/kg i.v.	(day −5) (3 and 6 ​min) 0.25, 0.5, 1, 2, 4, 8, 24, 48, 72, 96, 168, 240, 336, 504, 672, 1008, 1344, 1680, 2016, 2352
Tigolaner, emodepside & praziquantel	10	0.148 ​ml/kg topical	(day −5) 0.5, 1, 2, 3, 4, 6, 8, 24, 32, 48, 72, 96, 168, 240, 336, 504, 672, 1008, 1344, 1680, 2016, 2352, 2688, 3024, 3193
Study 2			
Tigolaner, emodepside & praziquantel	8	0.37 ​ml/kg topical	(day −5) 0.5, 1, 2, 3, 4, 6, 8, 24, 32, 48, 72, 96, 168, 240, 336, 504, 672, 1008, 1344, 1680, 2016, 2352, 2688, 3024, 3193
Tigolaner, emodepside & praziquantel	8	0.74 ​ml/kg topical	(day −5) 0.5, 1, 2, 3, 4, 6, 8, 24, 32, 48, 72, 96, 168, 240, 336, 504, 672, 1008, 1344, 1680, 2016, 2352, 2688, 3024, 3193
Study 3			
Tigolaner, emodepside & praziquantel	6	0.148 ​ml/kg topical	(pre-dose; 30 ​min) 1, 2, 4, 8, 24, 48 (day 7, 14, 21, 28)

*Note*: Treatment day: Day 0.

a Values in parentheses represent minutes.

Cats in studies 1 and 2 had daily individual social contact with their caretaker while in Study 3 cats were offered enrichment with toy balls. Room environment was monitored continuously in the studies, with a maximum temperature of 24 ​°C. Relative humidity ranged from 30% to 70%. Cages were cleaned daily with routine hygiene measures in place. Cats were fed once daily with a standard diet suitable for adult cats (Studies 1 and 2: commercial dry feed, Josera Kleinheubach Germany; Study 3: commercial dry feed, IAMS, Coevorden, Netherlands) and had *ad libitum* access to water.

All cats were individually identified by ear tattoo or electronic transponder. For studies 1 and 2, cats were randomized within sex using a randomized block design, except for some cats which were allocated to group based on their suitability for i.v. administration (Study 1) or to continue existing housing arrangement (Study 2). The studies were not blinded to treatment group.

In Study 1 where i.v. administration was performed, 6 animals per treatment group were treated with a slow manual i.v. bolus using a catheter placed in the *Vv. saphena medialis* or *Vv. saphena lateralis*, and suitable 1-ml single-use syringes. Catheters were placed immediately prior to administration and removed immediately afterwards. Tigolaner (in tetraglycol, C5H9O(OC2H4)nOH, CAS no. 31692-85-0) was administered at 1.5 ​mg/kg (volume: 70–80 ​μl), emodepside (in tetraglycol) was administered at 0.3 ​mg/kg (volume 40–70 ​μl) and praziquantel (in tetraglycol) was administered at 0.2 ​mg/kg (volume: 40–60 ​μl). Low dose rates were administered intravenously to ensure tolerance of an i.v. bolus. Where treatment was administered topically, a spot-on application was manually applied at the base of the head while the hair was divided with 2 fingers. In all studies, topical doses were calculated using individual BWs and the nominal content of the three AIs. The AIs were administered based on the licensed therapeutic dose of 14.5, 3 and 12 ​mg/kg BW for tigolaner, emodepside and praziquantel, respectively. Cats were restrained for approximately a minute following administration to aid spread of the applied formulation and to prevent any possible run-off. Table 2 provides details of the dosing of each of the three AIs in isolation *via* i.v. administration with topical Felpreva™. Cats were closely observed for 1 ​h after dosing and at least once daily thereafter.

General health observations (general demeanour, feed consumption, faeces consistency) were performed daily. Specific pre- and post-administration observations were performed before treatment, and 5 ​h and 29 ​h after treatment. Physical examinations were performed on study days −7, 14, 28, 39, 53 and 59/60. In studies 1 and 2, BWs were measured on study days −3, 28, 53 and 59/60, whilst in Study 3 BWs were measured on study days 1, 7, 14, 21 and 28. In studies 1 and 2 haematological and clinical biochemistry tests were performed at the beginning and end of the kinetic studies.

Blood samples of ∼0.5 ​ml (Studies 1 and 2) and 1.0 ​ml (Study 3) were collected into EDTA K2E tubes from the *Vv. cephalica antebrachii* or another suitable vein. Sampling times are shown in Table 2. Plasma was harvested following centrifugation (10 ​°C, 3220× *g* for 10 ​min) and subsequently stored frozen at −18 ​°C.

Urine and faeces were collected from cats in Study 3 for analysis of excretion of the AIs. Sampling days per cat were study days 1, 2, 7, 14, 21 and 28. Cats were kept in stainless steel cages with a litter box for the collection of total urine and faeces. The total volume of urine was determined, thereafter 2 aliquots (A ​+ ​B) of ∼5 ​ml urine were taken, collected in clear tubes and stored in a freezer set to maintain −18 ​°C. Faecal samples were weighed and stored in a freezer set to maintain −18 ​°C.

### 2.3 Analysis

#### 2.3.1 Pharmacokinetic analytical method

The methods were validated according to “Guidance for Industry: Bioanalytical Method Validation, U.S. Department of Health and Human Services, Food and Drug Administration”, Center for Drug Evaluation and Research (CDER), Center for Veterinary Medicine (CVM), May 2018 and “European Medicines Agency (EMA): Guideline on Bioanalytical Method Validation” EMEA/CHMP/EWP/192217/2009, 1 February 2012.

A very high specificity resulted from the HPLC separation in combination with MS/MS (tandem mass spectrometry) detection. No signals/peaks interfering with the detection of the analytes were observed in extracts of untreated control samples. Apparent concentrations of all analytes in control samples were below 0.3× ​limit of quantification (LOQ).

Analysis of all samples was performed after finalisation of the biological part of the study. The plasma samples were deproteinised by mixing 100 ​μl of plasma with 900 ​μl of a precipitation mixture of 0.040 ​g ammonium acetate in 100 ​ml water plus 0.1 ​ml formic acid and 600 ​ml acetonitrile containing the internal standards praziquantel-cyclohexyl-d_11_and [^13^C_2_H_6_] tigolaner and subsequent centrifugation. Analysis of the AIs tigolaner, emodepside, and praziquantel was conducted by using High Performance Liquid Chromatography with an Agilent Zorbax Eclipse Plus C18 Rapid Resolution, 2.1 ​× ​50 ​mm, 1.8 ​μm column, water/formic acid (1000/0.120, v/v) ​+ ​10 ​mMol/l ammonium formate and methanol/formic acid (1000/0.120, v/v) ​+ ​10 ​mMol/l ammonium formate as mobile phase (0–0.5 ​min at 90/10 v/v, gradient to 0/100 v/v at 3–3.5 ​min and gradient to 90/10 v/v at 4–5 ​min) at 60 ​°C with a flow of 0.6 ​ml/min. Detection was performed by Tandem Mass Spectrometry (HPLC-MS/MS) using a Sciex API 5500 mass spectrometer in the positive ionisation mode. Quantification of the samples was achieved by use of calibration curves (linear or quadratic, 1/× ​weighted) obtained by mixed matrix matched standards (containing the internal standards) in the range from 0.07 to 200 ​μg/l for tigolaner and emodepside and from 0.07 to 100 ​μg/l for praziquantel. The correlation coefficients were ≥ 0.9985. Recovery (accuracy) of fortified samples was a mean of 100–106% for each of the three AIs with mean relative standard deviation (RSD ​= ​precision) between 4.5% and 11.0%. The lower limit of quantification was 1.0 ​μg/l for tigolaner, 0.2 ​μg/l for emodepside and 0.1 ​μg/l for praziquantel in Study 1, whilst in subsequent studies the limit of quantification was 1.0 ​μg/l for the three AIs.

Urine samples were prepared for analysis by mixing 100 ​μl urine with 900 ​μl solvent mixture (as described above), containing labelled tigolaner and praziquantel as internal standards, and then centrifuged. Urine was analysed for the active substances tigolaner, emodepside and praziquantel by HPLC with a YMC Triart Phenyl, 2.1 ​× ​50 ​mm, 1.9 ​μm column, water/formic acid (1000/0.120, v/v) ​+ ​10 ​mMol/l ammonium formate and methanol/formic acid (1000/0.120, v/v) ​+ ​10 ​mMol/l ammonium formate as mobile phase (0–0.5 ​min at 70/30 v/v, gradient to 0/100 v/v at 3–3.5 ​min and gradient to 70/30 v/v at 4–5 ​min) at 50 ​°C with a flow of 0.6 ​ml/min, and MS/MS detection using a Sciex API 6500 mass spectrometer in the positive ionisation mode. Quantification was performed using matrix-matched standards (including the internal standards for tigolaner and praziquantel) in the range as described above (linear or quadratic, 1/× ​weighted, correlation coefficients ≥ 0.9991). Recovery, assessed using fortified samples, was a mean of 109% with RSD 4.7% for tigolaner, 109% with RSD of 4.4% for emodepside and 109% with RSD of 5.1% for praziquantel. The lower limit of quantitation was 1 ​μg/l.

Faecal samples were extracted by mixing 1 or 5 ​g faeces with 15 or 40 ​ml acetonitrile containing the internal standards. The mixture was ultrasonicated and shaken by means of an overhead shaker and then centrifuged. The supernatant was transferred into a flask and the residue extracted again with 5 or 30 ​ml extraction solvent, shaken, and centrifuged. The extracts were combined and filled up to 20 or 100 ​ml with extraction solvent containing the internal standards, then filtered. The extract was analysed for the active substances tigolaner, emodepside and praziquantel by HPLC-MS/MS using a Sciex API 5500 mass spectrometer under the same conditions as described for urine above. Quantification was performed using matrix-matched standards (including the internal standards for tigolaner and praziquantel) in the range from 0.4 to 75 ​μg/l, corresponding 8–1500 ​μg/kg in faecal samples (linear or quadratic, 1/× ​weighted, correlation coefficients ≥ 0.9995). Recovery, assessed using fortified samples, was a mean of 93% with RSD 5.1% for tigolaner, 95% with RSD of 5.9% for emodepside and 94% with RSD of 7.1% for praziquantel. The limit of quantitation was 10 ​μg/kg for each analyte.

#### 2.3.2 Pharmacokinetic analysis

PK and statistical evaluations were performed using the standard software Phoenix 64 (WinNonlin®, version 8.1; Pharsight Corporation (a Certara Company), Mountain View, California, USA). Separate evaluations were performed for the different AIs and study groups. Calculations comprised descriptive statistics on individual concentration data, PK analysis, and descriptive statistics on derived PK parameters (e.g. geometric mean (GM) and geometric standard deviation, minimum and maximum, 95% confidence interval). Plasma concentration-time-profiles were plotted individually and as geometric mean (± geometric standard deviation) curves by AI or study group. Graphic presentation was done using Prism version 8 (GraphPad Software, San Diego, CA, USA).

PK evaluation of the derived plasma concentrations were performed on the observed concentrations and planned sampling times (if actual times did not deviate outside the permitted time window) using non-compartmental methods. Each AI and treatment were evaluated separately. The software calculated a full set of PK parameters automatically.

The software selected the time points used for the terminal phase elimination rate constant calculation (λ_z_-calculation) automatically using the “best fit” approach calculated by means of log-linear regression. All parameters were derived from individual animal data sets.

Plasma drug concentrations analysed as being below the quantification limit (< LLoQ) were always entered into the system as “missing”. Relative data set weight was always set to 1.

#### 2.3.3 Calculation of topical bioavailability

In Study 1, the plasma exposure of the AIs tigolaner, emodepside and praziquantel were derived for Felpreva® after a single topical treatment at the therapeutic dose rates of 14.5 ​mg tigolaner, 3.0 ​mg emodepside and 12.0 ​mg praziquantel/kg BW. The plasma exposure was compared to the plasma exposure after i.v. dosing using single ingredient reference items and dose rates of 0.1× ​(tigolaner), 0.667× ​(emodepside), and 0.03× ​/0.0167× ​(praziquantel) the therapeutic dose rates due to the low tolerance of AIs when administered as an i.v. bolus.

The parallel study design did not allow calculating individual bioavailability. The group geometric mean dose normalized total exposure area under the curve extrapolated to infinity (AUCinf/D) was used instead to calculate a mean topical bioavailability/AI (F) using the following equation:

*F*abs ​= ​AUC_inf_/D, spot-on/AUC_inf_/D, i.v.

#### 2.3.4 Profiling of the pharmacokinetic characteristics of tigolaner, emodepside and praziquantel

The basic plasma PK characteristics of the active AIs tigolaner, emodepside and praziquantel were profiled after i.v. dosing based on at least the following parameters: Clearance (Cl), V, extrapolated concentration at time of dosing (C0), area under the curve until the last concentration above LoQ (AUC_last_), AUC_inf_, dose normalized AUC_last_ and AUC_inf_ (AUC_last_/D, AUC_inf_/D), mean residence time (MRT), and half-life. The statistical group mean estimates and suitable statistical parameters describing the distribution (scattering) were provided.

#### 2.3.5 Nonlinear mixed effects model building and evaluation of repetitive dosage of tigolaner

The changes in plasma concentration of tigolaner over time after a single spot-on administration were analysed using the stochastic expectation maximization (SAEM) algorithm implemented in Monolix Suite 2021R2 (Lixoft, Antony, France). We determined the individual values of pharmacokinetic parameters *post-hoc* using the mean of the full posterior distribution. The model was written as described earlier by Sheiner & Ludden (1992) and adopted to veterinary settings (e.g. Pelligand et al., 2016; Wang et al., 2019):

y_ij ​= ​F(φ_i,t_(ij)) ​+ ​G (φ_i, t_ij, β) ​× ​ε_ij

ε_ij ​∼ ​N (0, σ^2), φ_i ​= ​h(μ,η_i, β_i)

φ_i ​= ​μ ​× ​e^(η_i), η_i ​∼ ​N(0,Ω,ω^2)

j ​∈ ​{1, …,η_i }, i ∈{1, …,N}

where yij is the observed Substance X concentration measured in individual i (N is the number of all individuals) at time tij, whereas j describes the individual sample times from 1 to ni. Function F(φi, tij) is the predicted drug concentration at time tij dependent on the vector of individual pharmacokinetic parameters φi. The term G (φi, tij, β) X εij is the residual error model of F(φi, tij) where εij is an independent random variable distributed in a standard normal distribution with mean 0 and variance σ2. Individual parameters belonging to the vector φi were modelled as a function of the mean population parameter values, μ, individual variability ηi, and individual covariates, βi. The random variable ηi was assumed to be normally distributed with mean value 0, variance-covariance matrix Ω and variance ω2. As a result, individual parameters φi are log-normally distributed. The final model was parametrized with clearance (Cl), volume of distribution (V), absorption rate constant (ka), and lag time (Tlag). Only 4 of 260 (1.5%) concentration-time data points represented values below the limit of quantitation (BLOQ); therefore, a separate handling of BLOQ data was not included in the model.

Model quality was assessed using a set of accepted graphic and numerical tools (Pelligand et al., 2016; Nguyen et al., 2017). Convergence of the SAEM algorithm was checked by inspection of the stability of parameter search and by the precision of parameter estimates. This was measured by the relative standard error (RSE) of the estimate as obtained by the Fisher Information Matrix. The condition number of the eigenvalues was assessed to check for over-parameterization. Standard goodness-of-fit (GOF) plots were used to assess the performances of the different models: individual fits, individual predictions *vs* observations, normalized prediction distribution errors (NPDE), and visual predictive check. Normality and independence of residuals were assessed using histograms, quantile-quantile plots, and autocorrelation of conditional weighted residuals. Normal distribution of the random effects was assessed using the Shapiro-Wilk test as well as by inspection of the full posterior distribution of random effects and residuals. For converging models with satisfactory GOF diagnostics, corrected Bayesian information criterion (BICc) and the precision of the model parameter estimates were used for final model selection. The BICc was selected over the Akaikeʼs Information Criterion (AIC) as it tends to favour more parsimonious models (Mould & Upton, 2013; Wang et al., 2019).

##### 2.3.5.1 Parameter correlation estimates

Visual inspection of η *vs* η values for pharmacokinetic parameter estimates and Pearson’s correlation tests were used to evaluate the choice of correlations between the parameters. Correlation of random effects was applied when correlation coefficients were estimated to be high, met the threshold for inclusion (*P* ​< ​0.05) and improved model performance. As recommended by earlier studies (Lavielle & Ribba, 2016; Pelligand et al., 2016), multiple samples from the posterior distribution obtained at the last SAEM iteration were preferred over the empirical Bayes estimates (EBEs) during the evaluation of parameter correlations.

##### 2.3.5.2 Simulation of a multiple-dose administration

After model selection and fit, the R 3.4.4 package *Simulx 3.3.0* (Monolix 2021R2) was used to simulate tigolaner plasma disposition kinetic profiles from final Monolix run files. First, Monolix file was exported to *Simulx* and used to visualize the entire distribution of predicted tigolaner concentration time courses in cats, following a single administration of 14.5 ​mg/kg as a spot-on. Second, a population with 1000 cats was simulated and a multidose treatment with different intervals between doses (56 days (not shown) and 91 days) and an observation period of 600 days and this population was calculated with the population parameters which were set in the Monolix-file.

#### 2.3.6 Analysis of clinical data

The results of the pre- and post-treatment physical examinations and assessments were evaluated but not statistically analysed. Body weights were summarized as arithmetic mean and standard deviation per measurement and fluctuations in BW during the in-life phase were calculated. Any adverse event observed was described and assessed for a relation to treatment. Haematology and clinical chemistry data were analysed by means of Advia® 120 Haematology System (Bayer Diagnostics, Tarrytown, USA). Clinical chemistry was determined using a reflection photometer (VetTest 8008, IDEXX GmbH, 55286 Wörrstadt, Germany).

## 3 Results

Key pharmacokinetic parameters of tigolaner, emodepside and praziquantel administered topically as Felpreva® and intravenously alone are presented in Table 3.

**Table 3:** Selected mean plasma pharmacokinetics derived for the three active ingredients in cats.

Ingredient	Dose rate mg/kg	T_1/2_ (h)	T_max_ (h)	C_max_ (μg/l)	AUC_last_ (mg∗h/l)	AUC_inf_ (mg∗h/l)	Cl_pred (l/h/kg)	Vz_pred (l/kg)
Tigolaner topical	14.5	568.2 (26.1)	297.0 (40.5)	1245.1 (35.6)	1516 (32.8)	1566 (32.9)	–	–
Emodepside topical	3.0	347.5 (29.1)	36.9 (215.8)	44.3 (60.9)	20.5 (38.7)	20.6 (38.4)	–	–
Praziquantel topical	12.0	237.8 (23.8)	4.8 (107.7)	47.5 (56.8)	3.6 (19.7)	3.7 (19.6)	–	–
Tigolaner i.v.^a^	1.5	515.4 (54.2)			298 (29.5)	300.1 (29.1)	0.005 (31.7)	4.00 (56.5)
Emodepside i.v.^a^	0.2	202.4 (27.4)			1.5 (80.6)	1.6 (74.4)	0.131 (71.6)	38.25 (89.5)
Praziquantel i.v.^a^	0.2	1.8 (94.8)			0.1 (33.3)	0.1 (32.8)	1.861 (32.2)	4.95 (142.2)

*Note*: Mean values are given as geometric mean and geometric coefficient of variation in parentheses.

*Abbreviations*: i.v., intravenously; T_1/2_, plasma half-life; T_max_, time from dosing to the maximum concentration; C_max_, peak drug plasma concentration; AUC, area under the concentration *versus* time curve: 0 -Tlast (from the time of dosing to the time to the last quantifiable concentration), 0-inf (from the time of dosing to infinity (by extrapolation)); Cl_pred, systemic clearance; Vz_pred, volume of distribution at steady-state.

a Actual mean dose rates applied: 1.62 ​mg tigolaner, 0.21 ​mg emodepside, 0.21 ​mg praziquantel per kg body weight.

### 3.1 Single dose characteristics

**Fig. 1:**
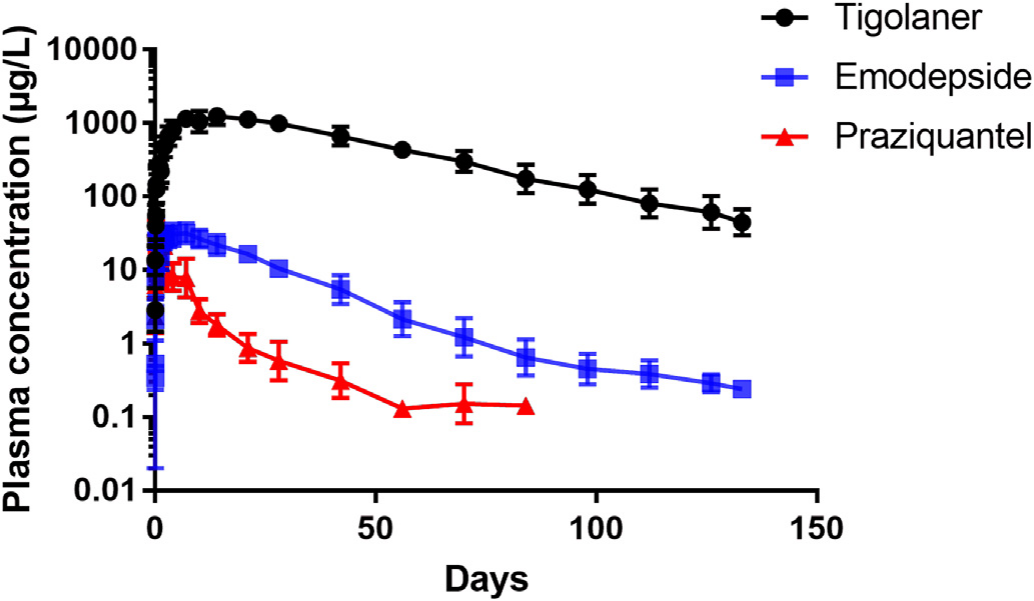
Mean plasma concentration profiles derived for Felpreva® (in log scale) following a single spot-on administration at the recommended treatment dose (Study 1). Geometric mean and geometric standard deviation of 10 cats treated topically with 0.148 ​ml/kg that equals to 14.5 ​mg/kg togolaner, 3 ​mg/kg emodespide and 12 ​mg/kg praziquantel.

The PK profile of each of the AIs when administered together as Felpreva® spot-on at a dose volume of 0.148 ​ml/kg showed an initial peak in plasma concentration followed by sustained levels over a prolonged period of time associated with distribution and elimination (Fig. 1). Following the single topical treatment, tigolaner had a calculated plasma exposure (AUC_inf_) of 1566 ​mg∗h/l, with the peak concentration of 1245 ​μg/l reached approx. 12 days (297 ​h) after dosing. Tigolaner was eliminated from plasma with a calculated half-life of almost 24 days (568 ​h). Emodepside peaked in plasma 37 ​h after dosing at a concentration of 44 ​μg/l. Total plasma exposure was 20.60 ​mg∗h/l and calculated half-life was approximately 14 days (348 ​h). Praziquantel showed a total plasma exposure of 3.69 ​mg∗h/l, with peak concentrations of 47 ​μg/l reached 5 ​h after dosing. It was eliminated from plasma at a mean half-life of 9.9 days (237 ​h) (Table 4, Table 5).

**Table 4:** Selected mean plasma pharmacokinetics derived after single dose equivalents of 1×, 2.5× and 5× test item.

Dose level	Dose (mg/kg)	C_max_ (μg/l)	T_max_ (h)	T_1/2_ (h)	AUC_inf_ (mg∗h/l)
Tigolaner					
1×	14.5	1245.1	297.0	568.2	1566
2.5×	36.25	2496.9	440.4	569.9	3308
5×	72.5	3574.2	526.6	563.2	5393
Emodepside					
1×	3.0	44.3	36.9	347.5	20.5
2.5×	7.5	71.3	47.8	329.2	36.1
5×	15.0	105.8	61.9	327.6	62.5
Praziquantel					
1×	12.0	47.5	4.8	237.8	3.7
2.5×	30.0	109.8	5.7	210.8	6.8
5×	60.0	176.3	4.7	193.4	14.2

*Note*: Mean values given as geometric mean.

*Abbreviations*: T_max_, time from dosing to the maximum concentration; C_max_, peak drug plasma concentration; T_1/2_, plasma half-life; AUC_inf_, area under the concentration *versus* time curve from the time of dosing to infinity (by extrapolation).

**Table 5:** Selected mean plasma pharmacokinetics and ratios derived after single dose equivalents of 1×, 2.5× and 5× test item.

Dose level	Dose (mg/kg)	C_max_/D (kg∗μg/l/μg) (Geo CV%) a	AUClast/D (h∗kg∗μg/l/μg) (Geo CV%) a	Ratio C_max_/D	Ratio AUClast/D
Tigolaner					
1×	14.5	0.092 (37.9)	103.16 (32.9)	–	–
2.5×	36.25	0.084 (137.8)	108.66 (142.5)	0.91	1.05
5×	72.5	0.053 (34.0)	78.5 (30.7)	0.58	0.76
Emodepside					
1×	3.0	0.015 (61.0)	6.79 (38.7)	–	–
2.5×	7.5	0.009 (71.4)	4.32 (44.2)	0.60	0.64
5×	15.0	0.008 (35.7)	4.5 (36.5)	0.53	0.66
Praziquantel					
1×	12.0	0.004 (57.2)	0.30 (19.9)	–	–
2.5×	30.0	0.003 (51.7)	0.19 (47.7)	0.75	0.63
5×	60.0	0.003 (39.8)	0.25 (22.2)	0.75	0.83

*Abbreviations*: C_max_/D, maximum observed concentration divided by dose; AUClast/D, area under the concentration *versus* time curve from the time of dosing to the last measurable concentration divided by the dose.

a Mean values given as geometric mean and geometric coefficient of variation in parentheses.

### 3.2 Bioavailability

Calculated bioavailability following topical application in comparison with i.v. administration was 57% for tigolaner, 90% for emodepside and 48% (first 24 ​h: 6%) for praziquantel. Pharmacokinetic profiling of the AIs after i.v. dosing was limited to 1.5 ​mg tigolaner, 0.2 ​mg emodepside and 0.2 ​mg praziquantel/kg BW due to limited tolerance of i.v. bolus administration. Pharmacokinetic parameters related to i.v. administration are shown in Table 3. Tigolaner showed a mean plasma exposure (AUC_inf_) of 300.12 ​mg∗h/kg with a volume of distribution of 4.0 ​l/kg and clearance of 0.005 ​l/h/kg. Mean emodepside plasma exposure was 1.61 ​mg∗h/l with a volume of 38.3 ​l/kg and clearance of 0.131 ​l/h/kg. Praziquantel showed a plasma exposure of 0.114 ​mg∗h/l and a volume of distribution of 4.95 ​l/kg and clearance of 1.861 ​l/h/kg.

### 3.3 Simulated repetitive administration of tigolaner every 91 days

**Fig. 2:**
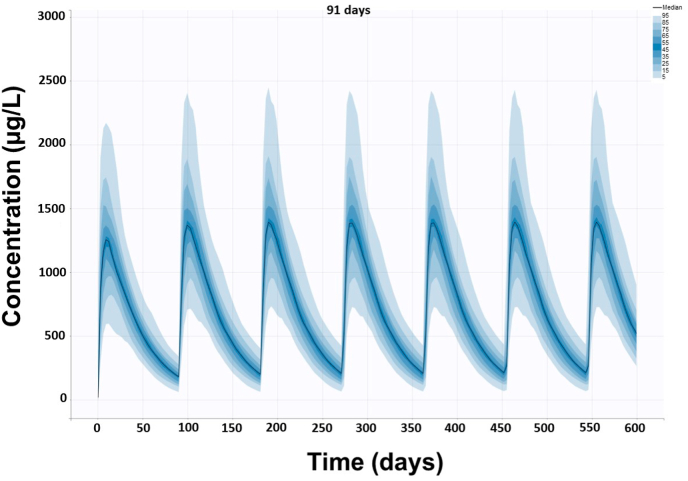
Simulated profile of repetitive administration of tigolaner every 91 days. Tigolaner data from Study 1 were analysed using the stochastic expectation maximization (SAEM) algorithm. After model selection and fit, tigolaner plasma disposition kinetic profiles were simulated from final Monolix run files. The Monolix file was exported to *Simulx* and used to visualize the entire distribution of predicted tigolaner concentration time courses in cats, following a single administration of 14.5 ​mg/kg as a spot-on. Second, a population with 1000 cats was simulated and a multidose treatment with different intervals between doses (91 days) and an observation period of 600 days was calculated.

Based on the kinetic data obtained in Study 1, a profile of repetitive topical administration of tigolaner every 91 days was simulated. As depicted in Fig. 2, there is a roughly a 10% increase of tigolaner concentration in plasma that reaches steady state after the third administration. Overall, the increase in plasma concentration as an indication of cumulation compared to single dose administration (Fig. 1) was modest. C_max_ increases from *c.*1250 ​μg/l to 1370 ​μg/l in steady state. Mean trough values increase from 180 ​μg/l to 200 ​μg/l in steady state.

### 3.4 Dose proportionality

**Fig. 3:**
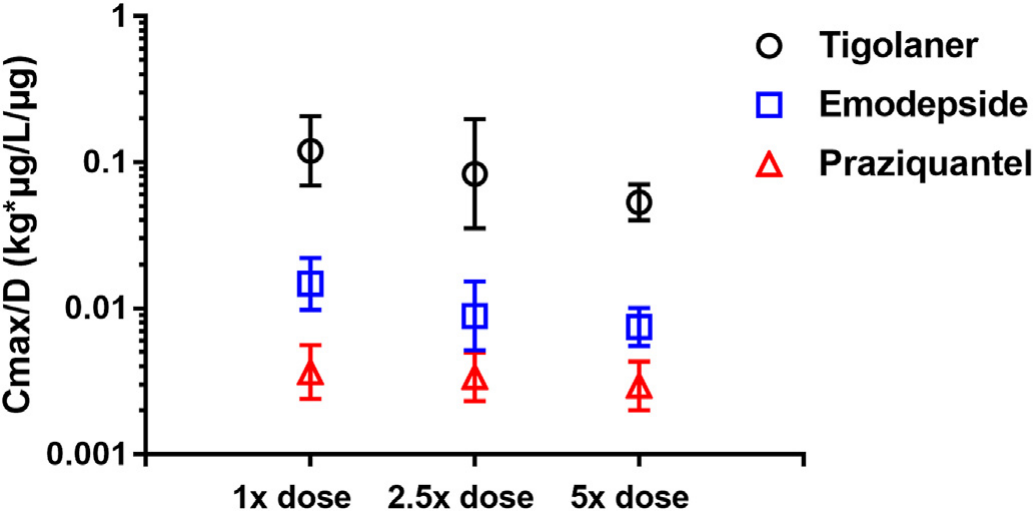
Extent of plasma exposure (C_max_/D) at different dose equivalents (data from studies 1 and 2).

**Fig. 4:**
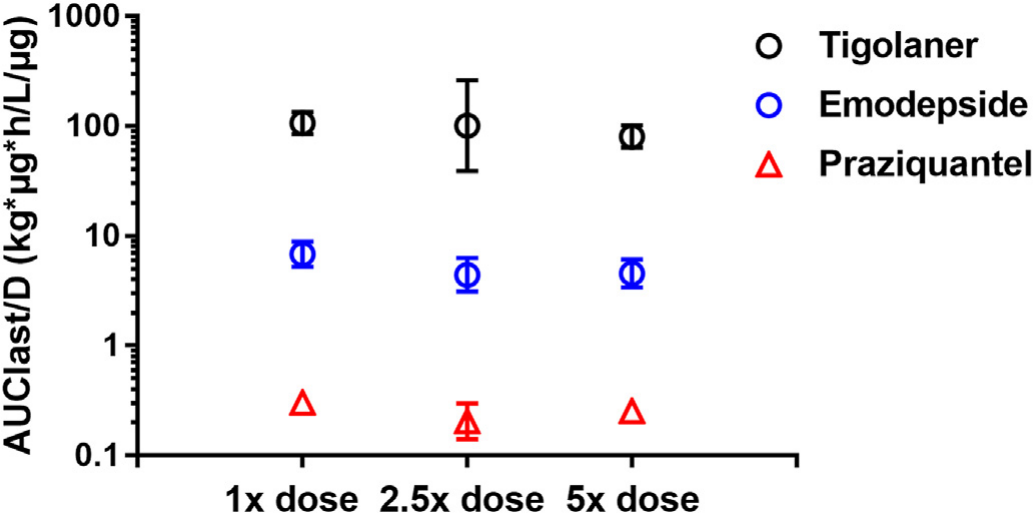
Rate of plasma exposure (AUC_last_/D) at different dose equivalents (data from studies 1 and 2).

The plasma PKs of the three AIs, tigolaner, emodepside and praziquantel, showed less than proportional increase in rate and extent with increasing dose rates from 1× to 2.5× and 5×, the target dose rate (Table 4, Table 5, Fig. 3, Fig. 4). The less than proportional increase was more obvious for C_max_/D compared to AUC_last_/D.

### 3.5 Excretion

After topical administration, tigolaner was mainly cleared *via* the faeces and approximately 55.5% in males and 53.2% in females of the administered dose was excreted after 28 days. Neglectable amounts of tigolaner was found in urine.

After topical administration, emodepside was mainly cleared *via* the faeces and approximately 56.7–70.5% of the administered dose was excreted after 28 days.

After topical administration, praziquantel was equally cleared *via* the faeces and *via* the urine for males and showed a slightly higher clearance *via* the faeces compared to the clearance *via* the urine for females. Approximately 1.38–1.47% of the administrated dose was excreted after 28 days.

In conclusion, tigolaner and emodepside seem to be poorly metabolized and mainly excreted *via* the faeces, whereas praziquantel undergoes substantial hepatic metabolism and only less than 2% are excreted equally *via* urine and faeces within 28 days of topical administration.

### 3.6 Application site and general health

All spot-on treatments were well tolerated by cats. The small volumes and doses used in the i.v. study were well tolerated. Even in the 2.5- and 5 times recommended dose group, none of the cats showed greater abnormalities in haematology, clinical chemistry or physical examination and were considered clinically inconspicuous over the observation period time of 133 days (data not shown).

## 4 Discussion

The present study reveals the pharmacokinetic profile of tigolaner, together with praziqantel and emodepside after topical administration to cats. The bis-pyrazole tigolaner shares many pharmacodynamic and pharmacokinetic characteristics with the isoxazolines (“laners”) including fluralaner, lotilaner, sarolaner and esafoxolaner (Kilp et al., 2016; Geurden et al., 2017; Toutain et al., 2018; Jacquot et al., 2021) that are licensed for cats. Comparable to the isoxazolines, tigolaner has a large volume of distribution, high level of protein binding and slow excretion, which provides it with the persistent characteristics required to protect cats from an established ectoparasite infestation and subsequent reinfestations. The observed long half-life and high volume of distribution of tigolaner translate into concentrations high enough to offer a three-months protection against fleas and ticks following topical application (Cvejić et al., 2022b; Mencke et al., 2023). As it emerges that fleas are prevalent all year round (ESCCAP, 2022), and for ticks a widespread and longer seasonal activity of *Ixodes ricinus* and *Dermacentor reticulatus* is observed e.g. within Europe, a sustained long-acting protection against ticks and fleas is recommended with the benefit of reduced vector-borne diseases (Bajer et al., 2022).

Felpreva® combines the established Profender® AIs, emodepside and praziquantel with tigolaner. The PK data demonstrate that also emodepside and praziquantel remain available at appropriate levels to exert their antiparasitic activity in the new combination. According to Profender® product information, emodepside reaches maximum serum concentrations of 32.2 ​± ​23.9 ​μg/l and praziquantel 61.3 ​± ​44.1 ​μg/l. T_max_ for emodepside is 3.2 ​± ​2.7 days after topical application and 18.7 ​± ​47 ​h for praziquantel. Both substances are eliminated from the serum with a half-life of 9.2 ​± ​3.9 days for emodepside and 4.1 ​± ​1.5 days for praziquantel (EMA, 2008). These data are in a comparable range as observed in the present study (Table 3) except for an extended half-life for praziquantel (9.9 days *vs* 4.1 days). However, even with a half-life of almost 10 days there is no issue with accumulation of praziquantel. When compared to a different spot-on formulation in cats (Nexgard® Combo, Boehringer Ingelheim, Ingelheim, Germany) the maximally achieved plasma concentrations for praziquantel seem a bit lower (107 ​± ​59 *vs* 47 ​± ​56.8 ​μg/l) but serum half-life seems longer with the formulation tested here (4.3 *vs* 9.9 days). However, the overall range is again similar (Jacquot et al., 2021).

The comparable pharmacokinetic profile of praziquantel and emodepside in Profender® and Felpreva® is reflected by similar clinical efficacy against parasites in naturally infected cats. In a randomized controlled study Felpreva® was proven to be as safe and effective as Profender® in the treatment of intestinal nematode, cestode and lungworm infections in cats under field conditions (Cvejić et al., 2022a), indicating that the pharmacokinetic properties of praziquantel and emodepside released from Felpreva® are reliable and that tigolaner shows only minor interference with absorption, distribution, metabolism, and excretion of praziquantel and emodepside in cats. Although the 2.5- and 5 times recommended dose administrations indicate a slightly less than proportional pharmacokinetic profile, particularly for AUC_last_/D, almost a linearity can be assumed. Individual cat observations across all treatment groups and for all studies indicate that Felpreva® administered at the recommended treatment dose (RTD) and up to 5× ​RTD was well tolerated. Due to the relatively long half-life of tigolaner, a simulation of repetitive topical administration (every 91 days) was performed. Although a slight cumulation was noticed, a steady state was reached after the third administration and thereafter for further administrations, the concentration of tigolaner should not increase further. Although the mean concentrations increased slightly from about 1250 ​μg/l to 1350 ​μg/l, this is far below concentrations observed at e.g. 2.5× and 5× recommended dose (Table 4) and these higher concentrations still were well tolerated by the cats. Thus, a repetitive administration every 91 days is considered safe.

## 5 Conclusions

The pharmacokinetic profile of emodepside, praziquantel and tigolaner, the three active ingredients of Felpreva® has been extensively studied. Pharmacokinetic characteristics of the novel ectoparasiticide tigolaner are described for the first time in cats. The large volume of distribution combined with long half-life of tigolaner accounts for its sustained activity against flea and tick infestations for up to three months after a single topical spot-on application with minimal effect on the pharmacokinetic profile of emodepside and praziquantel. Treatment with Felpreva®, including multiples of the recommended treatment dose rate, was well tolerated in cats.

## Funding

The study was funded by Bayer Animal Health GmbH as part of the required studies for registration for Felpreva® for marketing authorisation in Europe. The funders had no role in study design, data collection and analysis, decision to publish, or preparation of the manuscript.

## Ethical approval

The studies were designed in accordance with the standards of Good Clinical Practice (VICH Guideline 9). Cats were handled in compliance with the relevant Animal Care and Use/Ethics Committee approvals. Housing of cats complied with the Directive 2010/63/EU of the European Parliament and of the council of 22nd September 2010 on the protection of animals used for scientific purposes (including Annex III “Requirements for establishments and for the care and accommodation of animals”), the German animal protection act and the German welfare regulation for laboratory animals. Studies were performed in Germany (Studies 1 and 2) and the study design and experimental procedures had been approved by the responsible authorities (LANUV - Regional authority for nature, environment and consumer protection in North Rhine Westphalia). Study 3 was performed in the Netherlands and approved by the Central Authority for Scientific Procedures on Animals (CCD) as required by the Dutch Act on Animal Experimentation.

## CRediT authorship contribution statement

**Norbert Mencke:** Conceptualization, Funding acquisition, Writing – review & editing. **Wolfgang Bäumer:** Formal analysis, Writing – original draft. **Kristine Fraatz:** Investigation, Methodology, Formal analysis, Resources, Supervision. **Ralph Krebber:** Investigation, Methodology, Formal analysis. **Marc Schneider:** Formal analysis, Writing – review & editing. **Katrin Blazejak:** Writing – review & editing. All authors read and approved the final manuscript.

## Declaration of competing interests

The authors declare the following financial interests/personal relationships which may be considered as potential competing interests: Kristine Fraatz was an employee of Bayer Animal Health GmbH, Germany at the time while the studies reported here were conducted; today, an employee of Elanco Animal Health, Germany. Ralph Krebber is an employee of Bayer AG, Crop Science Division, Germany. Norbert Mencke, Katrin Blazejak and Marc Schneider are employees of Vetoquinol S.A., France.

## Data Availability

The data supporting the conclusions of this article are included within the article. Raw data generated in the study are confidential.
